# Genetic Polymorphisms in Human CX3CR1-Mediated Macrophage Dysregulation are Associated with the Worsening of Hearing Loss and Cochlear Degeneration After Noise Trauma: A Study in a Humanized Mouse Model

**DOI:** 10.21203/rs.3.rs-6578570/v1

**Published:** 2025-05-13

**Authors:** Dinesh Y. Gawande, Sree Varshini Murali, Shriti S. Thakur, Emma J. Nicolaisen, Lyudmila Batalkina, Astrid E. Cardona, Tejbeer Kaur

**Affiliations:** Creighton University; Rutgers, The State University of New Jersey; Rutgers, The State University of New Jersey; Rutgers, The State University of New Jersey; Creighton University; The University of Texas at San Antonio; Rutgers, The State University of New Jersey

**Keywords:** Macrophage, Fractalkine, CX3CR1, Polymorphism, Hearing loss, Noise trauma, Cochlea, Hair cell, Ribbon synapse, Inflammation

## Abstract

Sensorineural hearing loss (SNHL) is characterized by cochlear inflammation, macrophage activation, and degeneration of hair cells, synapses and neuron. Macrophage-mediated inflammation in the damaged cochlea is regulated via CX3CR1-CX3CL1 signaling, where the fractalkine ligand CX3CL1 serves as a chemotactic and calming signal for macrophage activation. Furthermore, disrupted CX3CR1-CX3CL1 signaling in CX3CR1-KO and CX3CL1-KO mice leads to reduced macrophage numbers, exacerbated inflammation and loss of hair cells, ribbon synapses and neurons in the damaged cochlea. Notably, ~ 25% of the human population carry single nucleotide polymorphisms (SNPs) in the CX3CR1 gene, CX3CR1^I249/M280^, which results in a receptor with lower binding affinity for CX3CL1, while most individuals carry the common wild-type CX3CR1^V249/T280^ allele. Although these polymorphisms are associated with various CNS neurodegenerative disorders, their impact on SNHL, cochlear degeneration and the macrophage response remains largely unknown. Here, we used a humanized mouse model expressing human CX3CR1 SNPs *in lieu* of its murine counterpart to investigate the effects of I249/M280 polymorphisms on cochlear function and structure following noise trauma. Young CX3CR1 WT, CX3CR1 KO, and human CX3CR1^I249/M280^ mice of both sexes were exposed to a noise level of 93 decibel sound pressure for 2 hours at an octave band (8–16 kHz). Cochlear function was assessed prior to exposure and at 1 day and 2 weeks postexposure. Also, the densities of inner and outer hair cells, ribbon synapses and macrophages in Rosenthal’s canal were examined after two weeks of exposure and compared among the three genotypes. We found that at 2 weeks postexposure, hearing thresholds were elevated and input–output function was impaired in hCX3CR1^I249/M280^ and CX3CR1 KO, whereas mice carrying WT alleles showed functional recovery. A significant synaptic loss (~ 30%) in hCX3CR1^I249/M280^ and CX3CR1 KO mice was observed relative to those in WT, which exhibited synaptic repair. hCX3CR1^I249/M280^ resulted in an ~ 17% loss of outer hair cells, which correlated with reduced otoacoustic emissions in the basal cochlear region. Noise led to increased macrophage numbers in the spiral ganglion and lateral wall of the WT; however, this response was attenuated in the CX3CR1 KO and hCX3CR1^I249/M280^ strains. Additionally, macrophages from CX3CR1 KO and hCX3CR1^I249/M280^ mice presented altered morphology and increased CD68 expression and inflammation. Compared with those of mice carrying the CX3CR1 WT or KO allele, young hCX3CR1^I249/M280^ mice fostered under ambient noise presented early elevations in hearing thresholds at basal frequencies. Together, these findings reveal that human CX3CR1 variant-mediated macrophage dysregulation strongly correlates with worsening of hearing loss and cochlear degeneration after noise trauma. Our work proposes a novel immune-related genetic polymorphism that may aid in the identification of individuals with increased vulnerability to SNHL.

## BACKGROUND

Sensorineural hearing loss (SNHL) is the most prevalent sensory disorder caused by damage to inner ear structures. It affects 1.57 billion people of diverse ages worldwide, with heterogeneous etiologies and manifestations (Haile et al., 2021). SNHL can be permanent or temporary and can affect one or both ears. Various factors can cause SNHL, including genetics, noise trauma, ototoxic drugs, infections, physical trauma, and biological aging, significantly impacting daily communication and quality of life. At the cellular level, SNHL involves complex pathological changes in the cochlea, the hearing organ of the inner ear, including damage to sensory inner hair cells (IHCs) and outer hair cells (OHCs), loss of ribbon synapses, degeneration of peripheral afferent dendrites and axons, and loss of spiral ganglion neurons (SGNs). OHC loss occurs immediately after prolonged exposure to loud noise (Yang et al., 2004; Hamernik et al., 1984); however, studies in rodents and humans have shown that acute exposure to moderate noise can primarily and rapidly damage IHC ribbon synapses without significant hair cell loss (Kujawa and Liberman, 2009; Liberman and Kujawa, 2017). As a result of these changes, it can be di cult to hear soft sounds and di cult to understand speech in noisy environments, which can be associated with tinnitus (ringing in the ear), hyperacusis (increased sensitivity to sound), or dizziness.

Inflammation is a hallmark of the pathology of SNHL, and macrophages are considered important drivers of both cochlear inflammation and structural damage (Frye et al., 2017; Kaur et al., 2015) and of tissue repair responses (Kaur et al., 2019; Manickam et al., 2023). Macrophages are the major leukocytes or innate immune cells in normal and damaged cochleae. Tissue (cochlear) resident macrophages can be found in developing (Dong et al., 2018) and adult mouse cochleae (Hirose et al., 2005) as well as in the human cochlea (Liu et al., 2018; O’Malley et al., 2016). Under steady-state conditions, macrophages reside in the cochlear spiral lamina, spiral ganglion neurons, stria vascularis, spiral ligament, and spiral limbus and below the basilar membrane in the scala tympani, whereas the organ of Corti is mostly devoid of macrophages (Fuentes-Santamaría et al., 2017). Owing to their cochlear location, these cells display unique phenotypes and shapes that may influence the maintenance of normal physiology (O’Malley et al., 2016; Liu et al 2018 & 2019; Okayasu et al., 2020; Noonan et al., 2020). Furthermore, cochlear insults due to exposure to moderate or loud noise, infections, aminoglycoside- or cisplatin-induced ototoxicity, or the insertion of cochlear implants can activate resident macrophages and induce infiltration of macrophages along with other immune cells from the blood into the damaged cochlea (Hirose et al., 2005; Yang et al., 2015; Frye et al., 2017; Kaur et al., 2019). These macrophages express various markers, such as CD45, Iba1, F4/80, CD68, and CX3CR1 (Hirose et al., 2005; Yang et al., 2015), and interact with sensory hair cells (Kaur et al., 2015a; Liu et al., 2019) and SGNs (Kaur et al., 2015a), phagocytose cellular debris (Kaur et al., 2018), regulate inflammation (Hirose, Rutherford and Warchol, 2017; Hu et al., 2018), promote the survival of sensory cells (Sato et al., 2010, Kaur et al., 2015a, 2018, 2019, Manickam et al., 2023, Sung et al., 2024), promote IHC synaptic repair (Kaur et al., 2019, Manickam et al., 2023) and influence the recovery (Manickam et al., 2023) or elevation (Sung et al., 2024) of hearing thresholds in the damaged cochlea.

Approximately 95% of cochlear-resident macrophages constitutively express CX3CR1, also known as the fractalkine receptor (Hirose et al., 2005), and its ligand, fractalkine/CX3CL1, a chemokine, is expressed in mature SGNs and IHCs (Kaur et al., 2015a; Liu et al., 2018). Notably, loss of hearing and degeneration of sensory hair cells, IHC ribbon synapses and SGNs are exacerbated in mice lacking CX3CR1 (Sato et al., 2010; Kaur et al., 2015a, 2018, 2019). Moreover, disruption of the CX3CR1/CX3CL1 axis due to the absence of either CX3CR1 or CX3CL1 results in fewer macrophages in the sensory epithelium and SGNs after cochlear injury (Kaur et al., 2015a, 2018, Manickam et al., 2024). Together, these findings suggest that the intact CX3CR1/CX3CL1 signaling axis is a potential chemoattractant for macrophages and plays an otoprotective role in the damaged cochlea. Notably, in humans, there are two common non-synonymous single-nucleotide polymorphisms (SNPs) in the open reading frame of CX3CR1: (i) a guanine-to-adenine substitution that results in a valine-to-isoleucine (V**→**I) at position 249 (rs3732379) and (ii) a cystine-to-thymidine substitution that results in a threonine-to-methionine (T**→**M) substitution at position 280 (rs373238) (McDermott et al., 2003; Faure et al., 2000). Most individuals carry the common wild-type CX3CR1^V249/T280^ allele, whereas the variant CX3CR1^I249/M280^ is present in ~ 20–30% of the population. The two SNPs are in strong linkage disequilibrium, forming a common I249/M280 haplotype, referred to as CX3CR1^I249/M280^ (McDermott et al., 2003; Moatti et al., 2001). This mutant receptor is proposed to function as hypomorphic (partially dysfunctional) and as a dominant-negative allele, such that leukocytes expressing the variant are less responsive to CX3CL1 ligand, have functional loss of tissue adhesion and chemotaxis, and impair monocyte survival in humans (McDermott et al., 2003; Cardona et al., 2018; Collar et al., 2018). Variant CX3CR1^I249/M280^ is strongly associated with an increased risk for neurodegenerative disorders such as age-related macular degeneration (AMD) (Tuo et al., 2004; Messal et al., 2023), multiple sclerosis (Stojković et al., 2012), amyotrophic lateral sclerosis (ALS) (Lopez-Lopez et al., 2014), and greater neuronal loss in the postmortem tissue of patients with late-onset Alzheimer’s disease (Lopez-Lopez et al., 2018), as well as an increased risk for otitis media (Nokso-Koivisto et al., 2014). Nevertheless, the relationship between the human variant CX3CR1^I249/M280^ in SNHL and cochlear degeneration has not been directly addressed.

Accordingly, we comprehensively characterized cochlear function and structure in a humanized mouse model that expresses the I249/M280 variants of the human CX3CR1 receptor (hCX3CR1I249/M280) *in lieu* of its murine counterpart in response to moderate synaptopathic noise. Our findings reveal that human CX3CR1 variant-mediated macrophage dysregulation strongly correlates with augmented hearing loss and cochlear degeneration and propose a novel immune-related genetic polymorphism that may aid in the identification of individuals with increased vulnerability to sensorineural hearing loss.

## MATERIALS AND METHODS

### Animals

**Figure 1:**
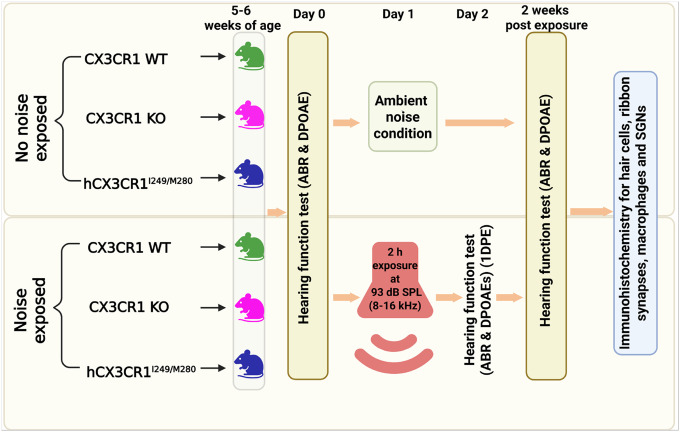
Schematic representation of the experimental protocol used to assess cochlear function and structure in CX3CR1 wild-type (WT), CX3CR1 knockout (KO), and humanized CX3CR1^I249/M280^ variants (hCX3CR1^I249/M280^) after noise trauma. Male and female mice at 5–6 weeks of age were used for the experiments. Mice from the three genotypes were exposed for 2 hours to a noise level of 93 dB SPL at an octave (8–16 kHz) band (Day 1) after the initial auditory testing. Auditory testing was performed at preexposure (day 0), 1 day (day 2) and 2 weeks postexposure. The mice were euthanized after the final auditory test, and histology was performed on the cochleae to immunolabel hair cells, ribbon synapses, macrophages and spiral ganglion neurons (SGNs). Mice from the three genotypes fostered under ambient noise conditions served as controls.

The studies used in-house bred CX3CR1-wild type (WT) (JAX stock number: 000664) and CX3CR1-knockout (KO) (JAX stock number: 005582) mice purchased from The Jackson Laboratory (Bar Harbor, Miane, USA). CX3CR1 WT and KO alleles were identified by genotyping as described previously by Kaur et al. (2015). Mice expressing human CX3CR1 variants (hCX3CR1^I249/M280^) were initially gifted by Dr. Astrid Cardona at the University of Texas, San Antonio (UTSA), and were bred, maintained and genotyped as described in Cardona et al. (2018). All the experiments involved male and female 5–7-week-old mice of the three genotypes. Table 1 shows the total number of mice used in the study, and Fig. 1 presents the study design. Efforts were made to minimize animal suffering and reduce the number of animals used. The animals were housed in autoclaved cages in groups of 5 under a 12 h light/12 h dark cycle and fed *ad libitum*. All aspects of animal care, procedures, and treatment were conducted according to the National Institute of Health guidelines and approved by the Animal Care and Use Committee of Creighton University and Rutgers University.

### Noise exposure

For noise exposure, we implemented the methodology described in our previous work (Manickam et al., 2023). Briefly, conscious and freely moving mice from each genotype were subjected to a 2-hour exposure to octave band noise (8–16 kHz) at a consistent intensity of 93 dB SPL. The exposure was performed within a sound-attenuating chamber (WhisperRoom) lined with acoustic foam. The mice were housed either singly or in pairs within modified compartmentalized cages from which food, water and bedding materials had been removed. These cages were arranged with a maximum of two units positioned directly beneath an exponential horn attached to a speaker (JBL). The acoustic stimulus was computer-generated via customized LabVIEW software interfaced with an audio card (Lynx E22), producing a filtered pure tone (8–16 kHz) that was subsequently amplified via a power amplifier (Crown Audio) connected to the horn speaker. Each exposure session was preceded by calibration via a quarter-inch condenser microphone (PCB) to verify the target sound pressure level (SPL), which varied by ± 1 dB across the compartments in the cage. The control groups consisted of unexposed age-matched mice of the same genotype.

### Auditory brainstem responses (ABRs)

We assessed auditory function via ABR measurements following established protocols (Manickam et al. 2023, 2024; Kaur et al., 2019). Briefly, the mice received intraperitoneal ketamine/xylazine anesthesia (100/20 mg/kg; 0.1 ml/20 g body weight) and were maintained at 37°C with ophthalmic ointment to prevent corneal drying. ABRs were recorded before exposure, 1 day after exposure, and 2 weeks after exposure via subcutaneous electrodes (reference: right pinna; active: vertex; ground: tail). Acoustic stimuli (5-ms tone pips, 0.5 ms cos2 rise–fall, 21/s, alternating polarity) were delivered via a TDT MF1 speaker positioned 10 cm from the right ear via a Tucker–Davis Technologies system. Responses were recorded at 12 kHz, filtered (300 Hz–3 kHz), and averaged (512–1024 per level) with BioSigRZ software, discarding artifacts exceeding 15 mV peak-to-peak voltage. The sound intensity decreased in 5 dB steps from 100 to 10 dB SPL. Thresholds at 5.6, 8.0, 11.2, 16.0, 22.6, and 32.0 kHz were determined by a single observer, who identified the lowest sound level producing a recognizable peak 1 waveform. Response validity was confirmed by increases in latency and decreases in amplitude with decreasing stimulus intensity. The ABR threshold was defined as the lowest SPL generating identifiable electrical response waves, with these values used to calculate the mean thresholds at each test frequency.

### ABR peak I input/output neural response

The methods followed those described by Kaur et al. (2019) and Manickam et al. (2023). Briefly, the ABR peak I component of the neural response was identified, and peak-to-trough amplitudes and latencies were computed through offline analysis of stored ABR waveforms. ABR peak I amplitude and latency versus stimulus level data were collected at 11, 22.6, and 32 kHz. The stimuli were presented in descending order from high to low sound levels (100 dB to 0 dB SPL), with 5 dB decrements from 100 dB SPL. Peak I amplitudes were measured from the estimated baseline prior to the response to the positive peak of peak I. Peak I latencies were measured as the time to peak I in milliseconds. ABR peak I amplitudes were analyzed at three time points: preexposure, 1 day postexposure, and 2 weeks postexposure.

### Distortion product otoacoustic emissions (DPOAEs)

As described in our previous reports (Manickam et al., 2023, Kaur et al., 2019), the mice were anesthetized as described above. We assessed cochlear outer hair cell function by measuring DPOAEs with acoustic stimuli delivered to the right ear via a custom-designed coupling insert. We generated DP grams across frequencies with f2 ranging from 5–40 kHz, maintaining a frequency ratio (f2/f1) of 1.2 and a level difference (L1-L2) of 10 dB. All recordings were conducted via BioSig RZ software. An observer blinded to the genotype performed DPOAE measurements at multiple timepoints: preexposure, 1 day postexposure, and 2 weeks postexposure.

### Immunohistochemistry

Two weeks after exposure, the mice were euthanized for histological analysis. We administered Fatal Plus (sodium pentobarbital) for deep anesthesia before performing intracardiac perfusion with phosphate-buffered 4% paraformaldehyde (PFA) (Electron Microscopy Sciences). After the temporal bones were isolated, they were postfixed in 4% PFA either for 15 minutes on ice (for synaptic immunolabeling) or 3–4 hours at room temperature (for hair cell, macrophage, and neuronal labeling). The samples were then rinsed three times in phosphate-buffered saline (PBS) for 15 minutes each and placed in 0.1 M ethylenediaminetetraacetic acid (EDTA) for decalcification, with durations of 12–15 hours for whole-mount microdissections and 3–4 days for cryosectioning.

For immunofluorescent visualization of both cochlear surface preparations and mid-modiolar frozen sections, we employed standard protocols. The tissues were washed with PBS (three times), followed by a 2-hour incubation at room temperature in blocking solution (5% normal horse serum in 0.2% Triton X-100 in PBS). We then incubated the cochleae overnight at room temperature with primary antibody cocktails. We identified hair cells via an anti-myosin 7a antibody (Proteus Biosciences, Cat. No. 25–6790, 1:500), whereas spiral ganglion neurons were visualized via a neuro lament 165 antibody (NF165, Developmental Studies Hybridoma Bank, Cat. No. 2H3C, 1:100) and HUD mouse (Santa Cruz Biotechnology, Cat. No. sc-28 299, 1:100). For synaptic components, we labeled IHC ribbon synapses at presynaptic zones with an anti-CtBP2 antibody (BD Biosciences, Cat. No. 612044, 1:200) and postsynaptic densities with an AMPA receptor GluA2 antibody (Sigma-Millipore, Cat. No. 6A2918; MAB397; 1:100). The macrophages were labeled with anti-CD45 (R&D Systems, Cat. No. AF114; 1:100), anti-IBA1 (Invitrogen, Cat. No. PA527436, 1:100) and anti-CD68 (AbD Serotec, Cat. No. MCA1957; 1:50) antibodies. Following incubation in primary media, the samples were rinsed 5 times in PBS and treated for 2 h at room temperature species-specific secondary antibodies conjugated to either DyLight 405 (1:500, Jackson ImmunoResearch Laboratories) or AlexaFluor-488, −546, −555, or −647 (1:500; Invitrogen). The tissue was mounted in glycerol:PBS (9:1) and coverslipped before confocal imaging. All tissue samples were batch processed using the same reagent solutions.

### Cellular imaging and analyses

We conducted fluorescence imaging using a Zeiss LSM 900 confocal microscope. For every cochlear sample, we acquired Z-series images at either 20x or 40x magnification with 1-micron z-step intervals or 63x magnification with 0.3-micron z-step intervals. Subsequent image processing and quantitative analyses were performed via a combination of 3D IMARIS image analysis software and ImageJ (Fiji) from the National Institutes of Health.

### Hair cell counts

We identified both inner and outer hair cells on the basis of their Myosin 7a immunoreactivity. Hair cell quantification was performed in distinct cochlear regions corresponding to specific frequency ranges: the apical (~ 4, 8, and 11 kHz), middle (~ 16, 22, 28 kHz), and basal (~ 32 and 45 kHz) regions. The results are presented as inner and outer hair cell counts relative to cochlear length.

### Macrophage counts and morphometric and activation analyses in the spiral ganglion

We evaluated macrophages in the Rosenthal’s canal or spiral ganglion by quantifying CD45- and IBA1-immunopositive cells in the basal, middle, and apical turns of cochlear midmodiolar sections. We acquired confocal z-stack images at 20x magnification with 1-micron step intervals and calculated the macrophage density per 1000 μm2 spiral ganglion per cochlear turn. For each cochlea, we analyzed a minimum of 5 midmodiolar sections to determine the number of macrophages. To characterize macrophage morphology, we adapted and modified the methods described in Young and Morrison (2018). Images were acquired at 20 μm z-stacks with 1 μm intervals between optical sections. The morphological analysis assessed changes in ramified structure, specifically examining processes (end point voxels) and branches (joint voxels) via the Analyze Skeleton (2D/3D) plugin in ImageJ (NIH). The sampling rate included approximately 15–20 macrophages from the apex, 7–14 from the middle, and 7–10 from the basal spiral ganglia, with values averaged across 5 cross-sections per cochlea per genotype. To determine the degree of macrophage activation, we colabeled CD45- and IBA-1-immunopositive cells with CD68 (a marker associated with macrophage proinflammatory and phagocytic phenotypes) and quantified the immunoreactivity intensity of each macrophage type via ImageJ, and the results are reported as the mean fluorescence intensity per macrophage.

### Ribbon synapse counts

We acquired confocal z-stack images via a 63x high-resolution oil-immersion objective, capturing three images per frequency region: the apex (~ 5–11 kHz), middle (~ 16–22 kHz), and base (~ 28–40 kHz) of each cochlea. Each z-stack encompassed the entire synaptic pole of ~ eight hair cells, with 0.3 μm z-step intervals, extending from the apical portion of inner hair cells to nerve terminals in the habenula perforata. For quantitative analysis, we utilized IMARIS software (version 9.9.0, Oxford) to perform 3D assessment of synaptic elements, including paired/juxtaposed and orphan presynaptic and postsynaptic puncta. We carefully adjusted thresholds across all channels to minimize background while preserving significant fluorescence signals and ensuring that distinct nearby fluorescent puncta remained separated. Juxtaposed synapses were identified as paired CtBP2 and GluA2 fluorescent surfaces. To determine synaptic density, we divided total synaptic counts by the number of surviving IHCs, and the results are expressed as the juxtaposed ribbon synapse density per IHC.

### Luminex assay

A Luminex assay was performed to detect the protein levels of cytokine chemokines, such as IL-1α, IL-1β, IL-2, IL-4, IL-6, IL-10, IL-12, IL-13, IL-15, IL-17, IL-18, IL-22, IL-23, IL-33, M-CSF, IFNα, IFNβ, IFNγ, TNF-α, CXCL1, CXCL2, CXCL10, CCL2, CCL3, CCL4, and CCL7, according to the manufacturer’s protocol. Five- to six-week-old mice of each genotype were exposed to 93 dB SPL noise at 8–16 kHz for 2 hours, or unexposed control mice served as controls. Animals were euthanized following phosphate-buffered saline intracardial perfusion; cochleae were isolated at 1 day after exposure. Magnetic beads coated with target antibodies were added to a 96-well assay plate. Cochlear protein lysates at a concentration of 50 μg were loaded in triplicate. Antigen standards, blanks, and sample lysates were incubated with magnetic beads at 600 rpm for 2 hours in the dark at room temperature. Following three washes, 25 μL of detection antibody was added to the beads, which were subsequently incubated in the dark for 30 min at room temperature. The beads were washed three times again, 50 μL of streptavidin-PE was added, and the mixture was incubated for 30 min in the dark at room temperature. Finally, the samples were resuspended in 120 μL of reading buffer, and the plate was read via a Luminex^™^ 100/200^™^ system (Thermo Fisher Scientific, FLEXMAP 3D). The acquired raw data were analyzed via the 5PL algorithm offered by Thermo Fisher Scientific (Procartaplex Analysis App available online). Raw data les from the instrument were fed into the application. From the panel of 26 cytokines, those that showed differential changes at 1 day postexposure with respect to preexposure as well as among the exposed genotypes are represented in the graph.

### Statistical analyses

Statistical analyses were performed via GraphPad Prism (version 10.4.2). The values are expressed as the means ± standard deviations (SDs) unless otherwise stated in the figure legends. To determine statistical significance, we selected appropriate tests for each parameter being examined, including t tests for two-group comparisons and one-way or two-way ANOVA for multigroup or multifactorial analyses. For significant main effects or interactions identified through ANOVA, we conducted suitable *post hoc* analyses to determine specific between-group differences. Comprehensive statistical details, including error representations, sample sizes, and experimental replication information, are provided in the results and corresponding figure legends. The results were considered statistically significant when the probability values (*p*) were less than or equal to the predetermined significance threshold of 0.05.

## RESULTS

### Human CX3CR1 polymorphisms are associated with exacerbated cochlear function after noise exposure.

**Figure 2:**
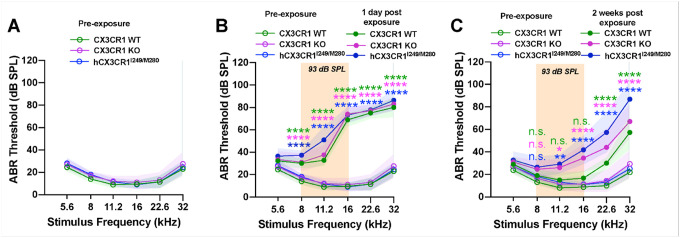
Poor recovery of ABR thresholds in hCX3CR1^I249/M280^ and CX3CR1 KO mice compared with that in CX3CR1 WT mice 2 weeks after noise exposure. A, ABR thresholds of CX3CR1 WT, CX3CR1 KO, and hCX3CR1^I249/M280^ prior to noise exposure. B, ABR thresholds of CX3CR1 WT, CX3CR1 KO, and hCX3CR1^I249/M280^ at 1 day postexposure. C, ABR thresholds of CX3CR1 WT, CX3CR1 KO, and hCX3CR1^I249/M280^ at 2 weeks postexposure. The data are presented as the means ± SDs. N=9–11 mice per genotype. ABR thresholds are statistically compared between preexposure and noise exposure for each genotype in B and C via two-way ANOVA and Tukey’s multiple comparisons test. Statistical comparisons (asterisks or n.s.) are color-coded on the basis of genotype, as indicated in the legends.

We previously reported that the absence of CX3CR1 on macrophages impairs the recovery of cochlear function after temporary threshold shift (TTS)-imparting noise (Kaur et al., 2019). To evaluate the effects of CX3CR1 variants on cochlear function after noise exposure, normal hearing age-matched CX3CR1 WT, CX3CR1 KO and hCX3CR1^I249/M280^ mice were exposed for 2 hours to a moderate noise level of 93 dB SPL at 8–16 kHz octave band. The primary goal was to determine how closely the expression of human CX3CR1 loss-of-function variants recapitulates the phenotype observed in CX3CR1-deficient mice. Auditory brainstem responses (ABRs) were recorded as a measure of neuronal activity of the ascending auditory pathway prior to (at 5 weeks of age, P35) and at 1 day and 2 weeks after exposure (at 7 weeks of age, P49). ABR thresholds in unexposed CX3CR1 WT, CX3CR1 KO and hCX3CR1^I249/M280^ mice at 5 weeks of age were not significantly different from each other at any of the 6 test frequencies (Fig. 2A, p = 0.9155, two-way ANOVA). One day after noise, all three genotypes presented elevated ABR thresholds relative to those of unexposed mice at test frequencies between 8 and 32 kHz, with an average minimum shift of ~ 17 dB SPL at 8 kHz and a maximum shift of ~ 60 dB SPL at 16 and 22.6 kHz (Fig. 2B). Although the ABR thresholds of the three genotypes were not significantly different from each other for any test frequency at 1 d after exposure, by 2 weeks after exposure, the recovery of thresholds were significantly better in CX3CR1 WT than in hCX3CR1^I249/M280^ and CX3CR1 KO at four test frequencies: 11.2, 16, 22.6 and 32 kHz (Fig. 2C, Table 2).

**Figure 3:**
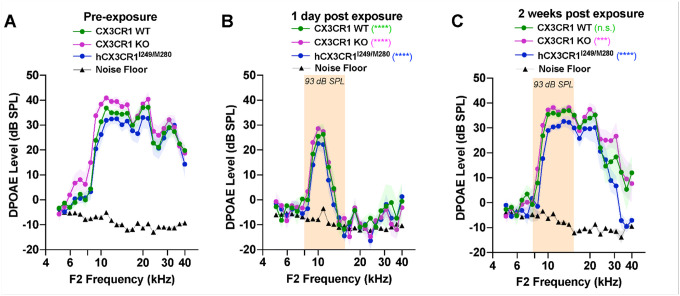
DPOAE levels remain reduced in hCX3CR1^I249/M280^ and CX3CR1 KO than in CX3CR1 WT 2 weeks after noise exposure. A, DPOAE levels in CX3CR1 WT, CX3CR1 KO, and hCX3CR1^I249/M280^ mice prior to noise exposure. B, DPOAE levels in CX3CR1 WT, CX3CR1 KO, and hCX3CR1^I249/M280^ at 1 day postexposure. C, DPOAE levels in CX3CR1 WT, CX3CR1 KO, and hCX3CR1^I249/M280^ at 2 weeks postexposure. The data are presented as the means ± SDs. N=6–8 mice per genotype. DPOAEs were statistically compared between preexposure and noise-exposed samples for each genotype in B and C via two-way ANOVA and Bonferroni’s multiple comparisons test. Statistical comparisons (asterisks or n.s.) are color-coded on the basis of genotype, as indicated in the legends.

Outer hair cell (OHC) integrity was assessed by measuring distortion product otoacoustic emission (DPOAE) levels prior to and at 1 day and 2 weeks after noise exposure. Figure 3A shows similar DPOAE levels in unexposed CX3CR1 WT, CX3CR1 KO and hCX3CR1^I249/M280^ mice at 5 weeks of age (p = 0.9893, two-way ANOVA). DPOAE levels were significantly reduced at 1 day after exposure than in unexposed mice for each genotype, with no significant difference among the three genotypes (p = 0.1214, two-way ANOVA) (Fig. 3B). By 2 weeks postexposure, DPOAE levels remained significantly depressed relative to pre-exposure levels in hCX3CR1^I249/M280^ and CX3CR1 KO (p < 0.0001 and p = 0.0001, respectively; two-way ANOVA), whereas CX3CR1 WT levels were not significantly different from preexposure levels (p = 0.0696, two-way ANOVA) (Fig. 3C, Table 3).

### Loss of outer hair cells in hCX3CR1 ^I249/M280^ after noise exposure.

**Figure 4:**
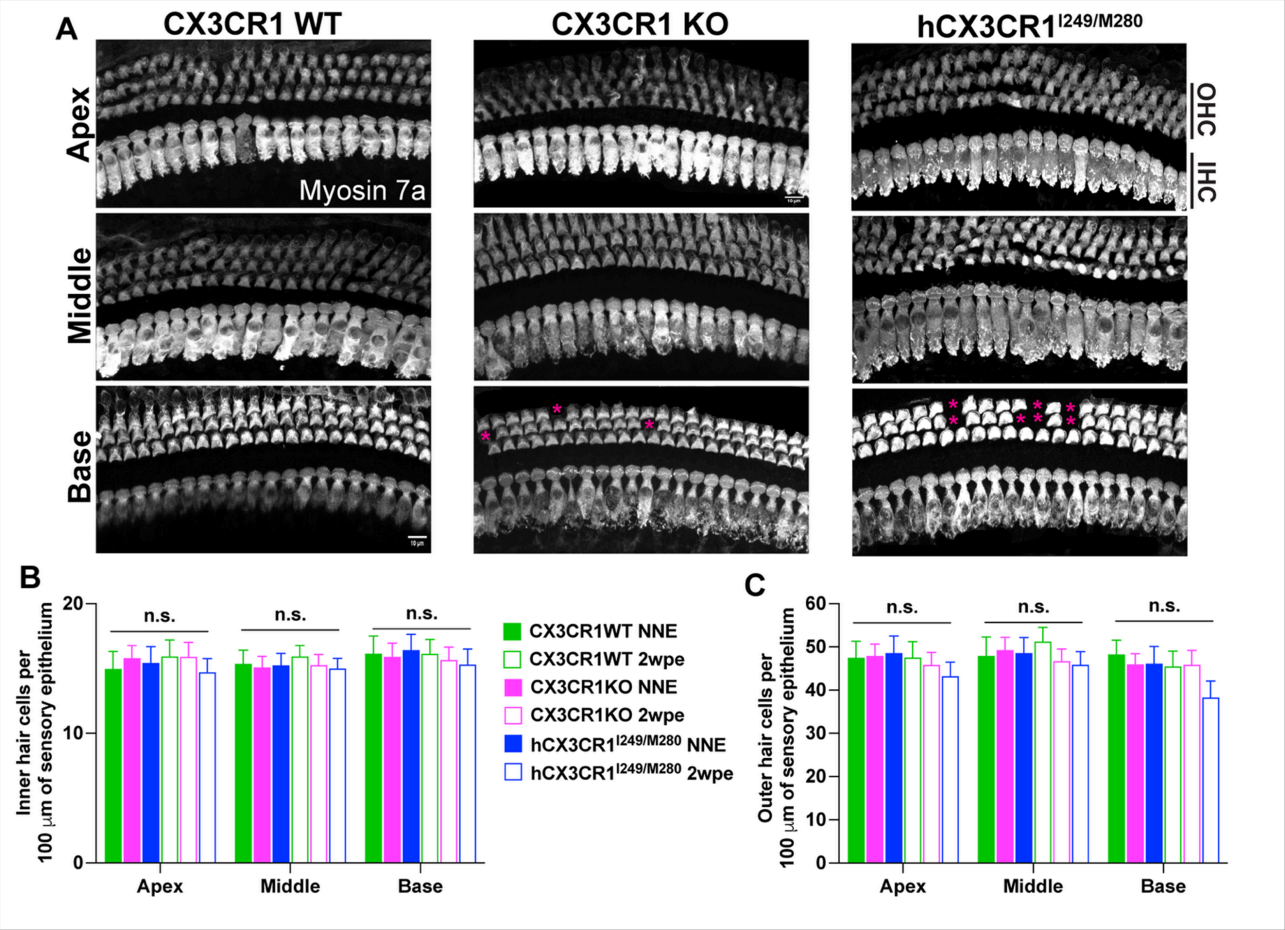
Outer hair cell loss in hCX3CR1^I249/M280^ after noise exposure. A, Representative confocal micrographs showing Myosin 7a-immunolabeled inner and outer hair cells in the apex, middle and base cochlear regions of CX3CR1 WT, CX3CR1 KO, and hCX3CR1^I249/M280^ at 2 weeks postnoise exposure. Magenta asterisks indicate the loss of OHCs in the base region of the cochlea of exposed CX3CR1 KO and hCX3CR1^I249/M280^ mice. Scale bar=10 μm in A. B, Quantification of inner hair cells (IHCs) per 100 μm of sensory epithelium. C, Quantification of outer hair cells (OHCs) per 100 μm of sensory epithelium. The data are presented as the means ± SDs. N=6–10 mice per genotype. Hair cell densities were statistically compared between no noise exposure (NNE) and 2 weeks postexposure (2 wpe) for each genotype per cochlear location in B and C via two-way ANOVA with Tukey’s multiple comparisons test. n.s., not significant.

Hair cell loss vulnerability increases in the absence of CX3CR1 in the damaged mouse cochlea (Sato et al., 2010, Kaur et al., 2019). To investigate whether human CX3CR1 variants also influence sensory hair cell integrity after noise, we conducted a quantitative analysis of hair cell populations across distinct cochlear regions. Hair cell counts were performed in the apical, middle, and basal turns, corresponding to different hearing frequency ranges. Under ambient noise conditions, quantitative assessment revealed no significant differences in either inner hair cell (IHC) or outer hair cell (OHC) numbers along the sensory epithelium between CX3CR1 WT, CX3CR1 KO, and hCX3CR1^I249/M280^. All three genotypes maintained comparable hair cell integrity across all cochlear regions in the absence of traumatic noise. Following exposure to noise, IHCs remained intact in all three genotypes throughout the cochlear spiral, whereas OHCs exhibited genotype-specific vulnerability to noise (Fig. 4A–C). Specifically, hCX3CR1^I249/M280^ mice demonstrated ~ 17% loss of OHCs in the base region of the cochlea, although the loss was not significantly different from that of unexposed hCX3CR1^I249/M280^ or exposed CX3CR1 WT and CX3CR1 KO mice (p = 0.2593, two-way ANOVA) (Fig. 4A and 4C). Nonetheless, these and above DPOAE data together imply that the presence of human CX3CR1 variants may increase the susceptibility of OHCs to dysfunction and damage from traumatic noise.

### Human CX3CR1 polymorphisms are associated with attenuated ABR peak I amplitudes and latencies after noise exposure.

**Figure 5:**
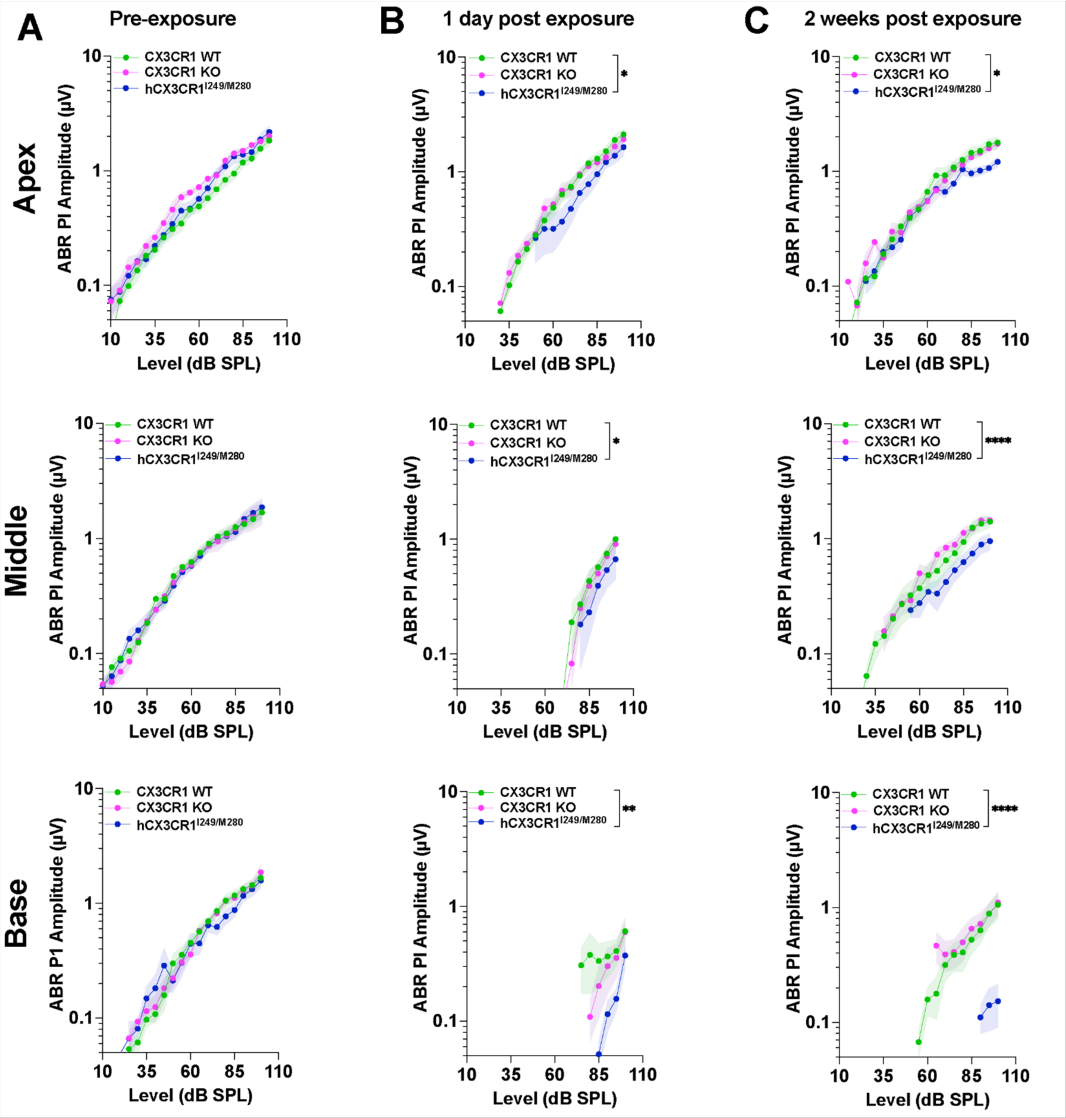
Poor recovery of ABR Peak I amplitudes in hCX3CR1^I249/M280^ and KO mice compared with WT mice after noise exposure. A, ABR PI amplitudes from the apex, middle and base cochlear regions in CX3CR1 WT, CX3CR1 KO, and hCX3CR1^I249/M280^ mice prior to noise exposure. B, ABR PI amplitudes from the apex, middle and base cochlear regions in CX3CR1 WT, CX3CR1 KO, and hCX3CR1^I249/M280^ at 1 day postexposure. C, ABR PI amplitudes from the apex, middle and base cochlear regions in CX3CR1 WT, CX3CR1 KO, and hCX3CR1^I249/M280^ at 2 weeks postexposure. The apex, middle and base correspond to stimulus frequencies of 11.2, 22.6 and 32 kHz, respectively. The data are presented as the means ± SDs. N=9–11 mice per genotype. Two-way ANOVA followed by Tukey’s or Bonferroni *post hoc* test was used to compare the PI amplitudes either between recovery time points per genotype or between genotypes per recovery time point. The asterisks shown in B and C represent the statistical comparison between CX3CR1 WT and hCX3CR1^I249/M280^ at the indicated time points postexposure. *p<0.05, **p<0.01, ****p<0.0001. Prior to exposure, the PI amplitudes were not significantly different among the genotypes at any cochlear location (p>0.05) (A). As expected, at 1 day postexposure, the amplitudes were significantly lower in all three genotypes in the middle and base regions of the cochlea and in the apical turn of CX3CR1 KO and hCX3CR1^I249/M280^ mice than in the preexposure amplitudes (p<0.0001) (B). However, the amplitudes in the apical turn of the CX3CR1 WT strain were comparable to those before exposure (p=0.3587) (B). At 2 weeks postexposure, the PI amplitudes were partially restored in the CX3CR1 WT but not in the CX3CR1 KO and hCX3CR1^I249/M280^ strains (C).

Along with the standard hearing tests (ABR thresholds and DPOAE levels), we also evaluated the ABR peak I amplitude and latency across the apical, middle, and basal cochlear regions, which corresponded to 11.2, 22.6, and 32 kHz, respectively. These parameters directly correlate with IHC ribbon synapse function, providing a functional assessment of synaptic integrity. Measurements were obtained at three time points: preexposure, 1 day and 2 weeks postexposure to 93 dB SPL noise for 2 hours. As expected, at 1 day postexposure, the amplitudes were significantly reduced in all three genotypes in the middle and base cochlear regions and in the apex of CX3CR1 KO and hCX3CR1^I249/M280^ than in the preexposure regions (p < 0.0001, two-way ANOVA), whereas the amplitudes in the apex of the CX3CR1 WT were comparable to the preexposure levels (p = 0.3587, two-way ANOVA) (Fig. 5A and 5B). Notably, the reduction in amplitudes in hCX3CR1^I249/M280^ was significantly greater than that in CX3CR1 WT at each cochlear region after exposure (Fig. 5B, see black asterisks). By 2 weeks postexposure, hCX3CR1^I249/M280^ and CX3CR1 KO exhibited persistently reduced amplitudes across the apex (p = 0.0169 for hCX3CR1^I249/M280^), middle (p < 0.0001), and basal (p < 0.0001) cochlear regions compared with those of CX3CR1 WT, which displayed partial restoration of amplitudes (Fig. 5C, two-way ANOVA).

**Figure 6:**
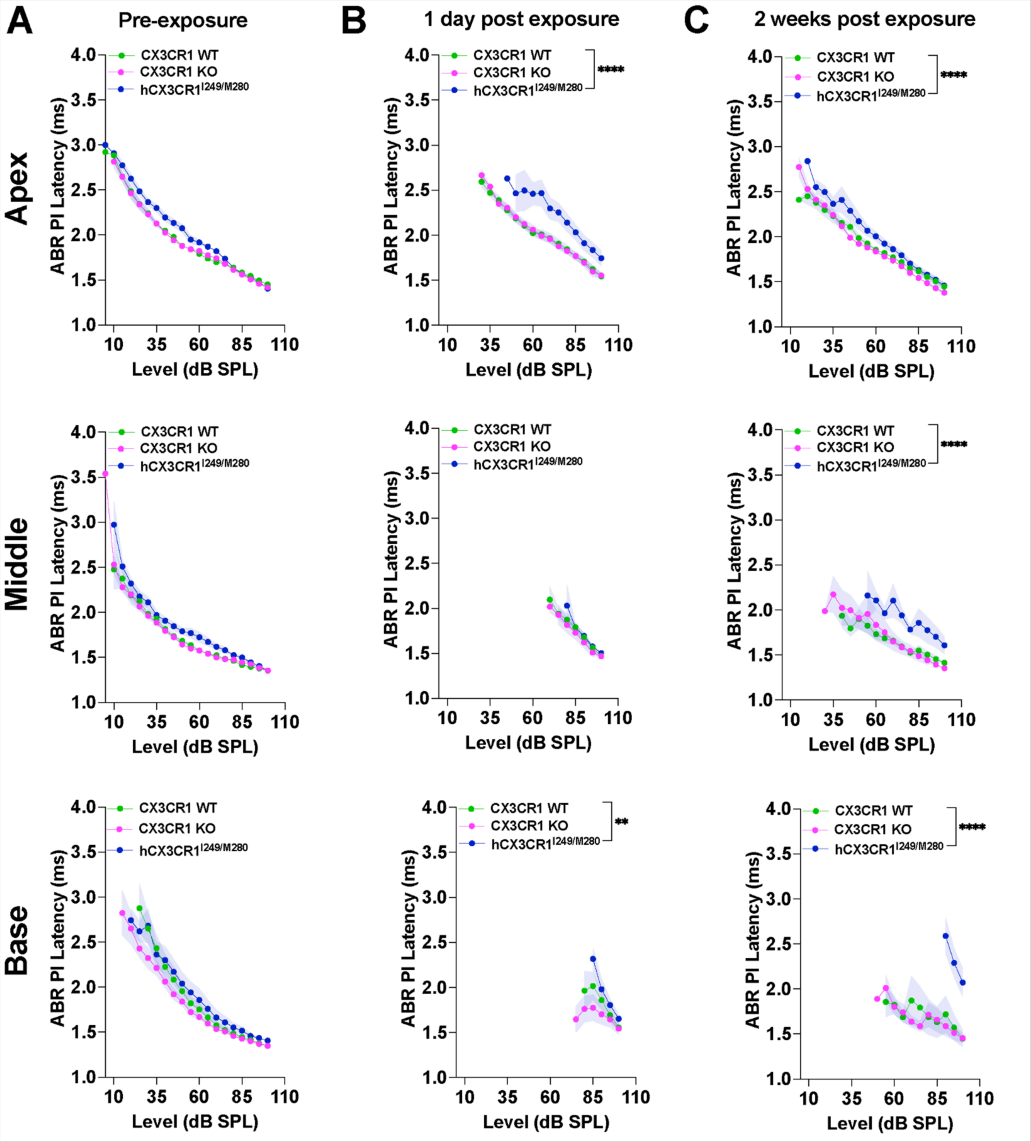
Poor recovery of ABR peak I latencies was observed in hCX3CR1^I249/M280^ mutants compared with wild-type and KO mice after noise exposure. A, ABR PI latencies from the apex, middle and base cochlear regions in CX3CR1 WT, CX3CR1 KO, and hCX3CR1^I249/M280^ mice prior to noise exposure. B, ABR PI latencies from the apex, middle and base cochlear regions in CX3CR1 WT, CX3CR1 KO, and hCX3CR1^I249/M280^ at 1 day postexposure. C, ABR PI latencies from the apex, middle and base cochlear regions in CX3CR1 WT, CX3CR1 KO, and hCX3CR1^I249/M280^ at 2 weeks postexposure. The apex, middle and base correspond to stimulus frequencies of 11.2, 22.6 and 32 kHz, respectively. The data are presented as the means ± SDs. N=9–11 mice per genotype. Two-way ANOVA followed by Tukey’s or Bonferroni *post hoc* test was used to compare the PI latencies either between recovery time points per genotype or between genotypes per recovery time point. The black asterisks shown in B and C represent the main statistical comparisons between CX3CR1 WT and hCX3CR1^I249/M280^ at the indicated time points postexposure. **p<0.01, ****p<0.0001. Prior to exposure, PI latencies were indistinguishable among the genotypes at any cochlear location (p>0.05) (A). As expected, at 1 day postexposure, latencies were significantly reduced in all three genotypes compared with preexposure latencies (p<0.0001). (B). At 2 weeks postexposure, the PI latency was partially restored in the CX3CR1 WT and CX3CR1 KO mice, with limited recovery in the hCX3CR1^I249/M280^ mice (C).

Similarly, ABR peak I latencies were also significantly reduced in all three genotypes at 1 day after exposure compared with preexposure latencies (p < 0.0001, two-way ANOVA), with a significantly greater reduction in hCX3CR1^I249/M280^ than in CX3CR1 WT in the apex and base cochlear regions (Fig. 6A and 6B, black asterisks). The latencies partially recovered in CX3CR1 WT and CX3CR1 KO whereas hCX3CR1^I249/M280^ presented limited recovery by 2 weeks post exposure and were significantly reduced when compared to CX3CR1 WT across apex (p < 0.0001), middle (p < 0.0001) and base (p < 0.0001) cochlear regions (Fig. 6C, two-way ANOVA).

### Inner hair cell ribbon synapses repair in CX3CR1 WT but not in CX3CR1 KO or hCX3CR1 ^I249/M280^ mice after noise exposure.

**Figure 7:**
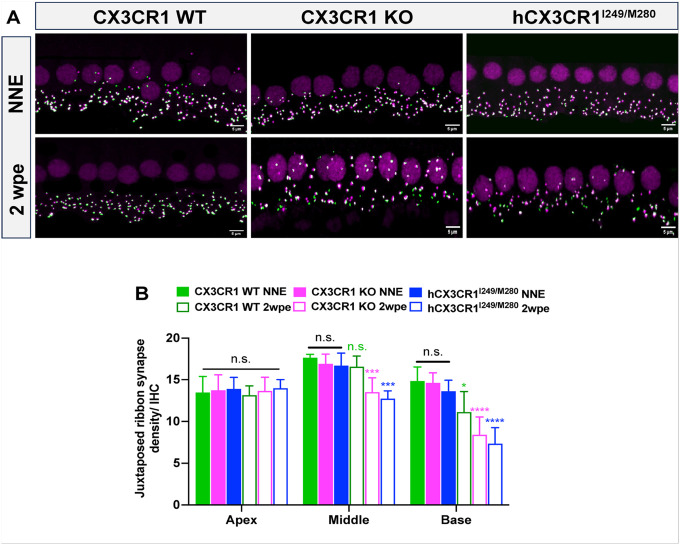
Synapses undergo repair in CX3CR1 WT but not in hCX3CR1^I249/M280^ and CX3CR1 KO after noise exposure. A, Representative confocal micrographs showing juxtaposed IHC ribbon synapses immunolabeled for the presynaptic marker CtBP2 and the postsynaptic marker GluA2 in the middle cochlear region of CX3CR1 WT, CX3CR1 KO, and hCX3CR1I249/M280 mice. Scale bar= 5 μm. B, Juxtaposed ribbon synapse density per IHC in CX3CR1 WT, CX3CR1 KO, and hCX3CR1I249/M280 mice at the apex, middle and base regions of the NNE and 2 weeks postexposure (2 wpe) cochleae. The data are presented as the means ± SDs. N= 7–8 mice per genotype. Synapse densities were statistically compared between NNE and 2wpe for each genotype in each cochlear region via two-way ANOVA with Tukey’s multiple comparisons test. *p<0.05, ***p<0.001, ****p<0.0001, n.s. not significant. Statistical comparisons (asterisks or n.s.) are color-coded on the basis of genotype, as indicated in the legends. The black n.s. represents comparisons between unexposed or exposed genotypes in each cochlear region.

We previously reported that IHC ribbon synapses undergo repair, which is impaired in mice lacking CX3CR1 after exposure to synaptopathic noise (Kaur et al., 2019). Therefore, we analyzed the IHC synapse density at 2 weeks after exposure in hCX3CR1^I249/M280^ to determine whether the expression of human CX3CR1 loss-of-function variants recapitulates the phenotype observed in CX3CR1-deficient mice. In the apical cochlear region, no significant difference in synapse density was detected between unexposed and exposed mice for each genotype (Fig. 7A and 7B, Table 4). However, in the middle and base cochlear regions, hCX3CR1^I249/M280^ and CX3CR1 KO resulted in significantly fewer synapses than in unexposed respective genotype as well as in exposed CX3CR1 WT (Fig. 7A and 7B, Table 4; p = 0.0002 and p = 0.0004, WT vs. hCX3CR1^I249/M280^ and p = 0.0038 and p = 0.0213, WT vs. KO, in the middle and base cochlear regions, respectively; two-way ANOVA). Collectively, the structural and functional synaptic data suggest that the dysfunctional CX3CR1 variant hCX3CR1^I249/M280^ is associated with worsening of IHC synaptic loss and impedes synapse repair after noise-induced hearing loss.

### Human CX3CR1 polymorphisms are associated with cochlear macrophage dysregulation and aggravated inflammation after noise exposure.

**Figure 8:**
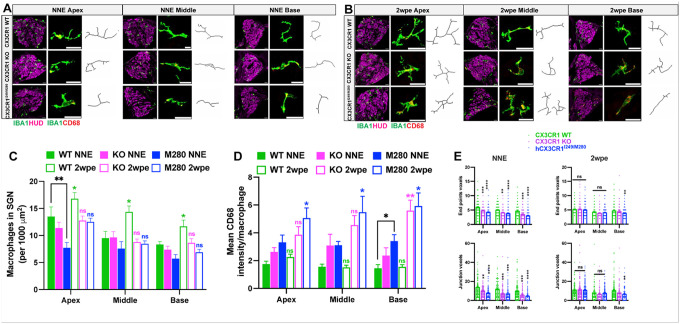
Macrophage dysregulation in hCX3CR1^I249/M280^ and CX3CR1 KO mice after noise exposure. A, Representative confocal micrographs from no noise-exposed (NNE) CX3CR1 WT, CX3CR1 KO, and hCX3CR1I249/M280 mice showing macrophages in the SGN (left panel), CD68-labeled macrophages (middle panel) and macrophage morphology (right panel) in the apex, middle and base regions of the cochlea. B, Representative images from 2 weeks postexposure (2wpe) CX3CR1 WT, CX3CR1 KO, and hCX3CR1I249/M280 mice showing macrophages in SGNs (left panel), CD68-labeled macrophages (middle panel) and macrophage morphology (right panel) in the apex, middle and base regions of the cochlea. Scale bar=20 μm for the left panel and 50 μm for the middle panels in A and B. C, Quantification of the macrophage density in the spiral ganglion revealed an increase in the number of macrophages in CX3CR1 WT mice after exposure but not in exposed CX3CR1 KO and hCX3CR1^I249/M280^ mice. D, Quantification of CD68 fluorescence intensity in macrophages in the spiral ganglion revealed greater CD68 expression in CX3CR1 KO and hCX3CR1^I249/M280^ than in CX3CR1 WT mice under naïve conditions and after noise exposure. E, Macrophage transformative index quantified as end point voxels (branch termini, upper panel) and junction voxels (branch points, bottom panel) shows altered or activated morphology of macrophages in CX3CR1 KO and hCX3CR1^I249/M280^ under naïve conditions and after noise exposure. The data in C, D and E are presented as the means ± SDs. N= 3 mice per genotype for NNE and 2wpe groups in C and D. N= ~30 macrophages per mouse per genotype for NNE and 2wpe groups in E. Two-way ANOVA followed by Tukey’s or Bonferroni *post hoc* correction was used to compare the data between NNE and 2wpe mice per genotype or among the three genotypes for the NNE and 2wpe groups in each cochlear region. Statistical comparisons (asterisks or n.s.) are color-coded on the basis of genotype, as indicated in the legends. The colored asterisks or n.s. shown in C, D and E represent the statistical comparison between NNE and 2wpe mice per genotype at the respective cochlear region. The black asterisks or n.s. shown in C, D and E represent the statistical comparison between CX3CR1 WT and CX3CR1 KO or CX3CR1 WT and hCX3CR1^I249/M280^ at the respective cochlear region for the NNE and 2wpe groups. *p<0.05, **p<0.01, *** p< 0.001, ****p<0.0001.

**Figure 9:**
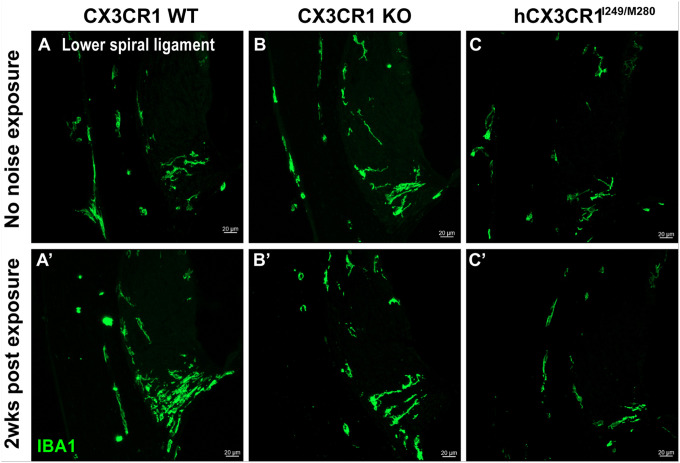
Macrophage numbers increased in the spiral ligament of CX3CR1 WT but not in those of hCX3CR1^I249/M280^ and CX3CR1 KO after noise exposure. A-C, Representative confocal images showing IBA1-immunolabeled macrophages in the lower spiral ligament of the basal turn of the cochleae of CX3CR1 WT (A), CX3CR1 KO (B), and hCX3CR1^I249/M280^ (C) mice prior to noise exposure. A’-C’ Representative confocal images showing increased numbers of IBA1-immunolabeled macrophages in the lower spiral ligament of the basal turn of the cochlea of CX3CR1 WT (A’) but not in those of CX3CR1 KO (B’) and hCX3CR1^I249/M280^ (C’) after 2 weeks of exposure to 93 dB SPL noise for 2 hours. Scale bar =20 μm. The images are representative of N=3 mice per genotype per noise exposure group.

Cochlear injury is associated with adaptive responses, including increases in the number of macrophages, which may regulate inflammation and influence structural damage and repair. We and others have demonstrated that CX3CR1 regulates the number of macrophages in the injured cochlea in a context-dependent manner; in the absence of CX3CR1, the number of macrophages either increases in the lateral wall after aminoglycoside ototoxicity (Sato et al., 2010) or decreases in the organ of Corti and spiral ganglia after selective hair cell ablation (Kaur et al., 2015a) or noise trauma (Kaur et al., 2018). The hCX3CR1^I249/M280^ variant disrupts CX3CL1-CX3CR1 signaling and leads to reduced macrophage adhesion and migration (McDermott et al., 2003; Cardona et al., 2018). Therefore, to study the effects of human CX3CR1 variants on the cochlear macrophage response after moderate noise, we assessed the density of CD45 (pan leukocyte marker)- and IBA1 (macrophage marker)-immunolabeled macrophages, specifically in the spiral ganglion, of CX3CR1 WT, CX3CR1 KO and hCX3CR1^I249/M280^ prior to exposure and at 2 weeks after exposure, and the densities were compared with those of the respective unexposed genotypes. Prior to exposure, the gross macrophage numbers in the spiral ganglion along the cochlea were comparable among genotypes, with the exception of slightly fewer macrophages in the apical ganglion of hCX3CR1^I249/M280^ than in WT alleles (Fig. 8A **(NNE, left panel)** and 8C, p = 0.0025; two-way ANOVA). Consistent with the findings of our previous studies, at 2 weeks after exposure, there was an ~ 25–40% increase in the number of macrophages in the spiral ganglion along the cochlea of CX3CR1 WT mice compared with those in unexposed WT controls (Fig. 8A–C, p = 0.05, 0.0198, and 0.0012 in the apex, middle and base, respectively; two-way ANOVA). In contrast, such an increase in the number of macrophages was not observed in the CX3CR1 KO and hCX3CR1^I249/M280^ strains, and the numbers were comparable to those of their respective unexposed counterparts (Fig. 8A–C; p = 0.9880 and 0.1413 in the apex, p = 0.9952 and 0.9076 in the middle and p = 0.8161 and 0.7778 in the base cochlear region of the KO and hCX3CR1^I249/M280^ strains, respectively; two-way ANOVA). Previously, Sato et al. (2010) reported that in the absence of CX3CR1, the number of macrophages in the lower spiral ligament of the lateral wall increases after aminoglycoside ototoxicity in mice. Therefore, we qualitatively examined the macrophage density in the lateral wall of CX3CR1 WT, CX3CR1 KO and hCX3CR1^I249/M280^ mice prior to exposure or 2 weeks after exposure. Notably, fewer macrophages were observed in the lower spiral ligaments of CX3CR1 KO and hCX3CR1^I249/M280^ mice than in those of CX3CR1 WT mice at 2 weeks postexposure (Fig. 9). These results corroborate our previous findings reported in Kaur et al., 2015a and 2018 and indicate that disrupted fractalkine signaling is associated with a weakened innate immune response after noise exposure.

In addition to macrophage density, gross morphology and CD68 (macrophage endosome and lysosome marker) expression were also assessed in the spiral ganglia to measure their activation. Analysis of CD68 expression, an indicator of phagocytic/reactive/inflammatory macrophages, revealed robust differences between genotypes under both naïve (unexposed) and noise-exposed conditions. Under naïve conditions, CD68 is expressed by all macrophages present in the spiral ganglion of CX3CR1 WT, CX3CR1 KO and hCX3CR1^I249/M280^. However, hCX3CR1^I249/M280^ and CXC3R1 KO macrophages presented elevated CD68 expression in the apex, middle and base spiral ganglia compared with that of CX3CR1 WT macrophages (Fig. 8A **(NNE, middle panel)** and 8D; p = 0.0295 WT vs. hCX3CR1^I249/M280^ in the base; two-way ANOVA). This CD68 expression was further elevated by ~ 2-fold in hCX3CR1^I249/M280^ macrophages in the spiral ganglia along the cochlear length after 2 weeks of exposure (Fig. 8B and 8D; p = 0.0181, 0.05, 0.0424 in the apex, middle and base, respectively, between unexposed and 2wpe hCX3CR1^I249/M280^, two-way ANOVA). Similarly, CD68 expression was also greater in exposed CX3CR1 KO macrophages in the base spiral ganglion than in unexposed KO macrophages (Fig. 8B and 8D, p = 0.0073 in the base, two-way ANOVA). In contrast to hCX3CR1^I249/M280^ and CX3CR1 KO macrophages, CX3CR1 WT macrophages present in the spiral ganglion presented no significant changes in CD68 expression at 2 weeks postexposure compared with unexposed CX3CR1 WT macrophages in any of the cochlear regions (Fig. 8B and 8D, p = 0.2269, > 0.9999, and > 0.9999 at the apex, middle and base, respectively; two-way ANOVA).

Furthermore, morphometric analysis revealed differences between genotypes under both naïve and noise conditions. Under naïve conditions, macrophages in the spiral ganglia of CX3CR1 WT mice displayed a ramified morphology characterized by long, thin, and branched processes. Quantitative assessment of process complexity measured by endpoint voxels (branch termini) and junction voxels (branch points) confirmed this ramified state (Fig. 8A **(NNE, right panel)** and 8E). In contrast, macrophages in the spiral ganglia of CX3CR1 KO and hCX3CR1^I249/M280^ mice displayed shorter processes and amoeboid cell bodies under naïve conditions (Fig. 8A **(NNE, right panel)** and 8E). After 2 weeks of noise exposure, the CX3CR1 WT macrophages maintained their ramified state, whereas the macrophages in the CX3CR1 KO and hCX3CR1^I249/M280^ mice exhibited less ramified morphology, with quantitative analysis confirming fewer processes and more amoeboid characteristics in hCX3CR1^I249/M280^ macrophages (Fig. 8B **(2wpe, right panel)** and 8E). These morphometric results indicate that cochlear macrophages in CX3CR1 KO and hCX3CR1^I249/M280^ exhibit altered morphology (reduced process complexity and more amoeboid characteristics) under both naïve and stressed (noise) conditions, suggesting a heightened reactive/inflammatory phenotype than WT macrophages.

**Figure 10:**
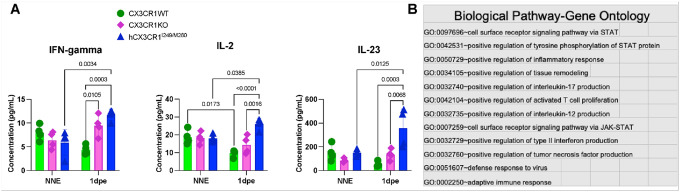
Cochlear inflammatory profile in WT, KO and hCX3CR1^I249/M280^ mice prior to and at 1 day after moderate synaptopathic noise exposure. A, Differential levels of the proinflammatory cytokines IFN gamma, IL-2, and IL-23 in WT, KO and hCX3CR1^I249/M280^ mice with no noise exposure (NNE) and at 1 day post exposure (1dpe). Following noise exposure, the cytokine levels are significantly greater in hCX3CR1^I249/M280^ than in the WT and KO alleles. The data are presented as the means±SDs. N=4 biological samples per genotype per recovery time point. Statistical significance was computed via two-way ANOVA and Tukey’s multiple comparisons test. B, Functional annotations of the upregulated cytokines in A via DAVID Bioinformatics (NIH).

Exposure to moderate synaptopathic noise is associated with the activation of macrophages (Kaur et al., 2019) and the upregulation of inflammation (Wu et al., 2020). Since intact fractalkine signaling attenuates the production of proinflammatory cytokines and decreases the activation of macrophages *in vitro* and *in vivo* following various inflammatory stimuli (Harrison et al., 1998; Ransohoff et al., 2007; Maciejewski-Lenoir et al., 1999; Garcia et al., 2000; Zujovic et al., 2000; Zujovic et al., 2001; Re and Przedborski, 2006; Lyons et al., 2009; **Mizutani et al., 2007**; Manickam et al., 2024) and inflammation is chronically upregulated in the absence of CX3CR1 in the damaged cochlea (Kaur et al., 2018), we examined whether the presence of hCX3CR1^I249/M280^ is also associated with heightened cochlear inflammation in response to moderate synaptopathic noise exposure. To address this, cochlear protein lysates were prepared from WT, KO, and hCX3CR1^I249/M280^ prior to and after 1 day of exposure and analyzed for cytokine levels via a bead-based Luminex assay. The data revealed that the levels of proinflammatory cytokines such as IFN-γ, IL-2, and IL-23 were distinctly elevated in hCX3CR1^I249/M280^ mice compared with WT mice after exposure (Fig. 10A). Notably, these differential cytokines are implicated in biological processes such as the activation of the inflammatory STAT1 pathway, adaptive T-cell proliferation and the production of proinflammatory cytokines such as IL-17 and IL-12 (Fig. 10B). Together, these findings indicate that disrupted fractalkine signaling through either the absence of CX3CR1 (KO) or loss-of-function mutation (hCX3CR1^I249/M280^) mediates macrophage dysregulation/dysfunction after traumatic noise exposure.

### Premature hearing loss in young hCX3CR1 ^I249/M280^ under ambient noise conditions.

**Figure 11:**
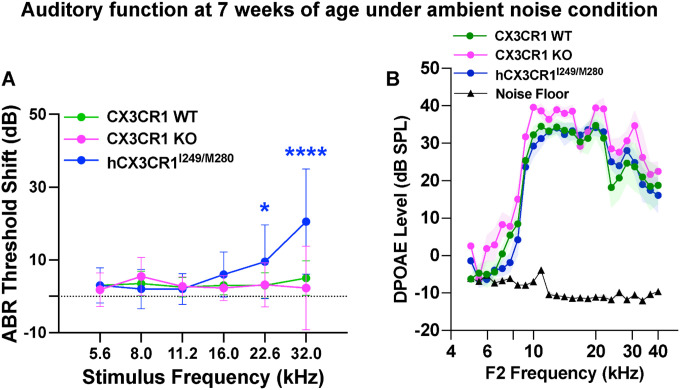
Elevated ABR thresholds at high frequencies in young hCX3CR1^I249/M280^ mice fostered under ambient noise conditions. A, ABR threshold shifts in CX3CR1 WT, CX3CR1 KO and hCX3CR1^I249/M280^ mice at 7 weeks of age when fostered under ambient noise conditions in the vivarium. N= 10–11 mice per genotype in A. ABR thresholds are significantly elevated at 32 and 22.6 kHz stimulus frequencies in hCX3CR1^I249/M280^ compared with baseline thresholds at 5 weeks of age and in age-matched CX3CR1 WT and CX3CR1 KO mice. ****p<0.0001, *p=0.045; two-way ANOVA, Tukey’s multiple comparison test. B, DPOAE levels in CX3CR1 WT, CX3CR1 KO and hCX3CR1^I249/M280^ mice at 7 weeks of age when fostered under vivarium ambient noise conditions. DPOAE levels are indistinguishable between genotypes (p=0.998; two-way ANOVA, Tukey’s multiple comparison test). N= 7–8 mice per genotype in B. Data are presented as the means±SDs in A and B.

Above, we found that mice expressing the human CX3CR1^I249/M280^ variants develop normally and do not exhibit any defects in gross cochlear function or structure in ambient noise at 5 weeks of age (P35) (Figs. 2–7). However, when these mice were tested again at 7 weeks of age (P49), we found that ABR thresholds were significantly shifted by ~ 20 dB at 32 kHz (p < 0.0001) and ~ 10 dB (p = 0.045) at 22.6 kHz (Fig. 11A, two-way ANOVA). Whereas the DPOAEs remained unaffected in these mice at P49 (p = 0.998) (Fig. 11B, two-way ANOVA). Since these mice are on the C57BL/6J background, we asked whether such progressive high-frequency hearing loss presumably reflects the effects of the Cdh23^753A^ allele known to be present in this background (Noben-Trauth et al., 2003). At 7 weeks of age, ABRs and DPOAEs remained unaffected in the CX3CR1 WT and KO unexposed mice, which were also on the C57BL/6J background (Fig. 11A, B). Hence, these data suggest that in addition to noise-induced hearing loss, the presence of human CX3CR1^I249/M280^ variants may also increase vulnerability to age-related hearing loss, likely due to the presence of dysfunctional macrophages.

## DISCUSSION

This study provides compelling evidence that genetic polymorphisms in the human fractalkine receptor CX3CR1 mediate macrophage dysregulation, which is associated with aggravated cochlear degeneration and hearing loss following acute exposure to moderate noise. Using a humanized mouse model expressing the common human polymorphic variant hCX3CR1^I249/M280^, we demonstrate that these genetic variants are functionally significant in the context of hearing physiology and pathology.

### CX3CR1 polymorphisms mediate macrophage dysregulation and aggravate cochlear inflammation

Our results revealed fundamental differences in the cochlear macrophage response among the three genotypes after noise. Mice carrying CX3CR1 WT alleles presented the expected increase in macrophage density within the spiral ganglion and lateral wall following noise, resulting in normal immune surveillance and repair functions (Hirose et al., 2005; Kaur et al., 2015). In contrast, this macrophage accumulation response was significantly attenuated in mice harboring the CX3CR1 KO or hCX3CR1^I249/M280^ alleles, suggesting a key role for endogenous fractalkine signaling in regulating the number of macrophages in noise-damaged cochleae. This finding corroborates our previous report in which CX3CR1 deficiency was associated with impaired macrophage accumulation in the cochlea after selective hair cell ablation in Pou4f3huDTR mice (Kaur et al., 2015a). Although mice expressing the hCX3CR1^I249/M280^ variant receptor exhibit CX3CR1 mRNA levels comparable to those of WT mice (Cardona et al., 2018), the blunted accumulation of macrophages in the variant cochlea is presumably attributed to the functional loss of tissue adhesion and chemotaxis of blood monocytes to CX3CL1 ligands (McDermott et al., 2003; Cardona et al., 2018). In addition, several reports have demonstrated that CX3CR1 signaling promotes the survival of mononuclear phagocytes such as blood monocytes and resident macrophages in the blood, kidney, liver and aorta (Collar et al., 2018), and CX3CR1 deficiency or dysfunctional CX3CR1 variant *CX3CR1-M280* is associated with decreased macrophage/monocyte survival due to impaired AKT and ERK activation following CX3CL1 ligand stimulation (Lionakis et al., 2013; Collar et al., 2018).

In addition to numerical differences, the morphological characteristics and activation states of cochlear macrophages were markedly altered in hCX3CR1^I249/M280^. Under both naïve and noise-damaged conditions, hCX3CR1^I249/M280^ macrophages displayed more amoeboid morphology and elevated CD68 expression than WT and KO macrophages, which is indicative of a proinflammatory phenotype (Mosser and Edwards, 2008; Chen et al., 2023). This macrophage phenotypic transformation is positively correlated with the differential upregulation of the proinflammatory factors IFN-γ, IL-2 and IL-23 in hCX3CR1^I249/M280^ mice after noise, which are involved in functions such as the activation of the STAT1 inflammatory pathway and T-cell proliferation. Overall, our data indicate that the presence of human CX3CR1 variants *CX3CR1-M280* results in macrophage dysregulation and inflammation, which may exacerbate cochlear degeneration and hearing loss after injury. Notably, our findings align with studies in the central nervous system showing that defective fractalkine signaling leads to abnormal microglial activation and aggravates neuroinflammation and tissue damage in neurodegenerative diseases (Cardona et al., 2006; Cardona et al., 2018; Mendiola et al., 2022).

### CX3CR1 polymorphisms exacerbate cochlear degeneration and hearing loss

Mice expressing the human CX3CR1^I249/M280^ variants presented a distinctive phenotype that closely resembled but was not identical to that of the CX3CR1-KO mice. Under ambient noise conditions, hCX3CR1^I249/M280^ displayed early onset of high-frequency hearing loss at 2 months of age compared with its WT and KO counterparts. This finding suggests that the human polymorphic variant not only results in partial loss of function but also potentially introduces novel detrimental effects beyond simple receptor deficiency. This aligns with previous characterizations of the I249/M280 variant as a hypomorphic, dominant-negative allele that impairs leukocyte tissue adhesion and chemotaxis (McDermott et al., 2003; Cardona et al., 2018). Following moderate noise exposure, both hCX3CR1^I249/M280^ and CX3CR1-KO mice failed to recover hearing thresholds at frequencies above 16 kHz, whereas thresholds recovered in WT mice. This differential recovery pattern mirrors previous findings of Kaur et al. (2019), who reported impaired recovery of cochlear function in CX3CR1-deficient mice after noise. Our results support these observations by revealing that human CX3CR1 variants confer similar functional deficits, highlighting the clinical relevance of these findings to human populations.

Perhaps the most significant finding of this study is the pronounced impact of hCX3CR1^I249/M280^ on IHC ribbon synapses. The CX3CR1 variant hCX3CR1^I249/M280^ was associated with significant synaptic loss in the middle (21%) and basal (32%) cochlear regions following noise, which was comparable to the synapse loss observed in CX3CR1-KO mice. However, the synapse density in the WT was similar to the unexposed density. These data indicate that normal fractalkine signaling is essential for synaptic maintenance and repair after noise injury, which is consistent with previous studies by Kaur et al. (2019) and Manickam et al. (2023). Intriguingly, while both CX3CR1-KO and hCX3CR1^I249/M280^ mice presented significant synaptic loss, we observed subtle differences in their ABR peak I amplitude and latency profiles, suggesting that the human CX3CR1 variants may introduce distinct pathophysiological mechanisms beyond simple receptor deficiency.

Interestingly, OHCs displayed genotype-specific vulnerability, whereas IHCs were largely resistant to the noise exposure paradigm employed in our study. Compared with WT and KO mice, hCX3CR1^I249/M280^ mice presented greater OHC loss in the basal region, which was positively correlated with reduced DPOAE levels in the same region. A study by Sato et al. (2010) reported increased OHC loss in CX3CR1-deficient mice after ototoxic injury. OHCs are particularly susceptible to metabolic stress and oxidative damage (Hirose et al., 2005), and their survival may depend on the proper resolution of inflammation following noise. The selective vulnerability of OHCs in hCX3CR1^I249/M280^ may reflect the combined effects of impaired macrophage-mediated resolution of inflammation and tissue repair and the dominant-negative properties of the variants. The attenuated accumulation of macrophages, along with their abnormal activation and heightened inflammatory environment observed in hCX3CR1^I249/M280^, likely exacerbates oxidative stress, thereby contributing to OHC degeneration.

### Clinical implications

The present findings have significant implications for clinical otology and audiology. Given that 20–30% of Caucasians carry the CX3CR1I249/M280 polymorphism (McDermott et al., 2003), our results suggest that a substantial portion of the population may have inherently greater susceptibility to noise-induced hearing loss. This genetic predisposition could explain some of the heterogeneity observed in clinical outcomes following noise exposure and potentially inform personalized hearing conservation strategies. Moreover, the accelerated age-related hearing loss at high frequencies observed in hCX3CR1^I249/M280^ suggests that this polymorphism may also contribute to presbycusis in humans. This finding aligns with the established associations between CX3CR1 variants and other age-related neurodegenerative conditions, such as age-related macular degeneration (Tuo et al., 2004), Alzheimer’s disease, and multiple sclerosis (Stojković et al., 2012). Our findings thus provide a potential immunological mechanism linking these seemingly disparate conditions through macrophage dysfunction. The association of hCX3CR1^I249/M280^ with both noise-induced and age-related hearing loss positions this genetic variant as a promising biomarker for identifying individuals at heightened risk for sensorineural hearing loss. Genetic screening for CX3CR1 polymorphisms could enable targeted interventions for susceptible individuals, such as enhanced hearing protection strategies and modified noise exposure guidelines. Furthermore, our findings provide a rationale for exploring therapeutic approaches targeting fractalkine signaling or macrophage modulation as potential treatments for sensorineural hearing loss.

### Limitations and future directions

While our study provides valuable insights, several limitations should be acknowledged. First, we focused on a specific noise exposure paradigm designed to induce cochlear synaptopathy and temporary threshold shifts. Future studies should explore the effects of the hCX3CR1^I249/M280^ variants under various graded noise conditions, including those causing permanent threshold shifts. Second, our analysis was limited to relatively young mice (up to 2 months of age). Extending observations to older mice would provide a more comprehensive understanding of how these polymorphisms influence the progression of age-related hearing loss and cochlear degeneration. Future investigations should explore the molecular mechanisms underlying the phenotypic differences between CX3CR1-KO and hCX3CR1^I249/M280^ mice. Transcriptomic and proteomic analyses of cochlear tissues and isolated macrophages would help identify downstream signaling pathways affected by CX3CR1 deficiency and variants. In addition, depletion of dysregulated macrophages in hCX3CR1^I249/M280^ cochleae will validate the causal role of these cells in the exacerbated functional and structural phenotypes observed in these mice. Finally, examining the heterozygous expression of the human variants would more accurately model the genetic condition in most human carriers and could reveal gene-dosage effects relevant to clinical outcomes.

### Conclusion

Our study provides the first direct evidence that human CX3CR1 polymorphisms significantly aggravate cochlear degeneration and hearing loss following noise exposure, with effects comparable to those of CX3CR1 deficiency. The underlying mechanisms involve dysregulated macrophage responses characterized by reduced recruitment and persistent inflammatory activation, leading to impaired synaptic repair and outer hair cell loss. These findings establish a novel immune-related genetic risk factor for sensorineural hearing loss and highlight the critical role of fractalkine signaling in regulating macrophage function during cochlear homeostasis and repair. By identifying genetic determinants of noise susceptibility, our work opens new avenues for personalized approaches to hearing conservation and potential immunomodulatory therapies for hearing loss.

## Figures and Tables

**Table 1:** Numbers, sex and age of the mice per genotype used in the study.

	CX3CR1 WT	CX3CR1 KO	hCX3CR1^I249/M280^
**Unexposed**	10 (5 M + 5 F)	9 (6 M + 4 F)	11 (6 M + 5 F)
**Noise exposed**	9 (5 M + 4 F)	10 (7 M + 3 F)	10 (5 M + 5 F)
**Postnatal age (weeks)**	P35–49 (5–7 weeks)	P35–49 (5–7 weeks)	P35–49 (5–7 weeks)

**Table 2:** ABR thresholds (dB SPL, mean ± SD) for test frequencies showing significant differences between CX3CR1 WT, CX3CR1 KO and hCX3CR1 I249/M280 2 weeks after 93 dB exposure^a^

	Tone frequency (kHz)			
	11.2	16	22.6	32
**CX3CR1 WT (N = 9)**	15.00 ± 6.61	16.66 ± 6.12	30.00 ± 5.00	57.22 ± 14.60
**CX3CR1 KO (N = 10)**	26.00 ± 7.37	34.50 ± 12.12	44.00 ± 16.46	67.00 ± 14.56
**hCX3CR**^**I249/M280**^ **(N = 11)**	29.09 ± 8.31	41.81 ± 12.70	57.27 ± 17.93	86.81 ± 15.21
**p (2-way ANOVA) CX3CR1 KO vs. CX3CR1 WT**	n.s.	0.0005	0.0131	n.s.
**p (2-way ANOVA) hCX3CR1**^**I249/M280**^ **vs. CX3CR1 WT**	0.0096	< 0.0001	< 0.0001	< 0.0001

(^a^See also Fig. 2.)

**Table 3:** DPOAEs (mean ± SD) in unexposed, or 2 weeks after 93 dB SPL in CX3CR1 WT, CX3CR1 KO and hCX3CR1 ^I249/M280 a^

	Unexposed	2wpe
**CX3CR1 WT**	21.40 ± 2.95	19.09 ± 3.02
**CX3CR1 KO**	24.93 ± 2.95	20.88 ± 3.19
**hCX3CR1** ^**I249/M280**^	19.28 ± 2.74	13.15 ± 3.20
**p (2-way ANOVA) CX3CR1 KO vs. CX3CR1 WT**	n.s.	n.s.
**p (2-way ANOVA) hCX3CR1**^**I249/M280**^ **vs. CX3CR1 WT**	n.s.	< 0.0001

(^a^See also Fig. 3.)

**Table 4:** Paired synapses (mean ± SD) per IHC in unexposed, or 2 weeks after 93 dB SPL exposure, CX3CR1 WT, CX3CR1 KO and hCX3CR1 ^I249/M280^ in the apical, middle, and basal cochlea^a^

	Genotype	Unexposed	2wpe	*p* (2-way ANOVA) CX3CR1 KO vs. CX3CR1 WT or hCX3CR1^I249/M280^ vs. CX3CR1 WT at 2 wpe
**Tonotopic region**				
**Apex**	**CX3CR1 WT**	13.44 ± 1.93	13.12 ± 1.12	
	**CX3CR1 KO**	13.71 ± 1.87	13.62 ± 1.65	n.s.
	**hCX3CR1** ^**I249/M280**^	13.87 ± 1.40	13.95 ± 1.04	n.s.
**Middle**	**CX3CR1 WT**	17.6 ± 0.43	16.52 ± 1.29	
	**CX3CR1 KO**	16.86 ± 1.19	13.48 ± 1.73	0.0006
	**hCX3CR1** ^**I249/M280**^	16.65 ± 1.52	12.71 ± 0.94	< 0.0001
**Base**	**CX3CR1 WT**	14.82 ± 1.69	11.85 ± 3.02	
	**CX3CR1 KO**	13.38 ± 3.24	8.38 ± 2.14	0.0356
	**hCX3CR1** ^**I249/M280**^	13.58 ± 1.35	7.31 ± 1.93	0.0097

(^a^See also Fig. 7.)

## Data Availability

All the Research data generated is provided within the manuscript.
